# Burn injury alters the intestinal microbiome’s taxonomic composition and functional gene expression

**DOI:** 10.1371/journal.pone.0205307

**Published:** 2018-10-05

**Authors:** Nadine Beckmann, Amanda M. Pugh, Charles C. Caldwell

**Affiliations:** 1 Department of Surgery, University of Cincinnati, Cincinnati, Ohio, United States of America; 2 Division of Research, Shriners Hospital for Children, Cincinnati, Ohio, United States of America; Klinikum rechts der Isar der Technischen Universitat Munchen, GERMANY

## Abstract

Burn patients have a high risk of sepsis-related mortality even after surviving the initial injury. Immunosuppression increases the risk of sepsis after burn injury, as does the disruption of the intestinal epithelial barrier, which allows the translocation of bacteria and bacterial products into the circulation. The integrity of the intestinal epithelial barrier is largely maintained by the intestinal microbiota. Burn injury has been reported to result in significant changes in the intestinal microbiome composition. In this mouse study, we confirm these taxonomic differences in a full-thickness scald injury model using CF-1 mice. For the first time, we also address alterations in functional gene expression of the intestinal microbiota after burn injury to assess the microbiome’s physiological capabilities for overgrowth and pathogenic invasion: 38 pathways were differentially abundant between the sham and burn injury mice, including bacterial invasion of epithelial cells and gap- and adherens junction pathways.

## Introduction

Burn patients, who survive the initial trauma, develop a state of relative immune compromise, making them highly susceptible to infections [1, 2]. Burn patients also suffer from a loss of endothelial and mucosal integrity [3]. In combination with the relative immune suppression the outcome is detrimental (massive tissue edema, bacterial translocation, multi-organ failure) and in some cases lethal [4, 5].

In healthy individuals, the gut microbiome protects against pathogenic microbes and helps maintain the intestinal epithelial barrier [6]. This commensal system, consisting of over 100 trillion microbes, also provides a number of other benefits to the host, i.e. metabolism and *de novo* synthesis of nutrients and assistance in immune development and function [7]. The gut microbiome is composed of between 300 and 1,000 species of bacteria. However, 99% of the total mass consists of only 40 species, among which three highly abundant phyla contribute the most to pathogen control and gut function: *Firmicute*, *Proteobacteria* and *Bacterioidete [8, 9]*. Changes to the microbiome result in a dysbiosis, that disrupts this regulatory environment, enabling low abundant and pathogenic populations (i.e. *Clostridium difficile*) to expand [10, 11]. Dysbiosis also contributes to the disruption of the intestinal barrier, enabling the translocation of bacteria and their products from the gut lumen to the mesenteric lymph nodes and on to the circulation [12]. Thus, the gut becomes a potential source of bacterial infections and sepsis after burn injury.

Several factors have been reported to affect the composition and biodiversity of the microbiome, including diet, environment, medication, infection/inflammation and burn injury itself [13–15]. On day one after burn injury, the gut is overgrown with gram-negative aerobic bacteria, including opportunistic pathogens [13]. We confirm this finding in a mouse model and expand the existing knowledge by analyzing gene expression of the whole microbiome to analyze their physiological capabilities for overgrowth and pathogenic invasion.

## Materials and methods

### Mice

Male CF-1 mice were obtained from Charles River Laboratories (Wilmington, MA) at five weeks of age and allowed to acclimate for one to two weeks prior to conducting experiments. All mice were housed in standard environmental conditions and had *ad libitum* access to pellet diet and water. All experiments are approved by the Institution Animal Care and Use Committee (IACUC number 08-09-19-01) of the University of Cincinnati.

### Scald burn injury

We used a scald burn injury model as previously described [16] Briefly, male CF-1 mice 6–8 weeks of age were randomized into two groups: scald and sham. Scald mice were anesthetized with 4.5% inhaled isoflurane in oxygen. Mice were placed in a template exposing their previously shaven dorsal surface and immersed in in 90.0°C water for 9 s. The scald procedure yields a 28% total body surface area burn (total surface area calculated based on the Meeh formula [17]. It is a full thickness, third degree, insensate lesion, as the destruction of the entire thickness of the dermis and its peripheral sensory endings was confirmed histologically [18]. The procedure typically has a mortality of less than 10%. Mice were subsequently resuscitated intraperitoneally with 1.5 mL sterile saline. Sham treated mice underwent the same procedure expect for the immersion in water. All murine experiments were performed between 8 AM and 1 PM. After injury, the mice are monitored to ensure that they wake up from the anesthesia and subsequently allowed to recover on a 42.0°C heating pad for 3 h. Mice are then returned to their home cage and monitored for any complications twice a day until the experiment is completed. No animal showed overt signs of morbidity that required euthanasia prior to the experimental endpoint.

### Sample collection and isolation

On post burn day 1 (PBD1), mice were euthanized with CO_2_ at a 30% fill rate and cecal fecal samples (100–200mg) were collected. Nucleic acid isolation was performed with the MoBio PowerMag Microbiome kit (Carlsbad, CA) according to the manufacturer’s guidelines and optimized for high-throughput processing. All samples were quantified via the Qubit Quant-iT dsDNA High Sensitivity Kit (Invitrogen, Life Technologies, Grand Island, NY) to ensure that they met minimum concentration and mass of DNA.

### Library preparation

Samples were prepared for sequencing with the Illumina Nextera XT kit and quantified with Quant-iT dsDNA High Sensitivity assays. Libraries were pooled and run with 100 bp paired-end sequencing protocols on the Illumina NextSeq 500 platform.

### Data analysis

#### Raw data processing

Host sequences were removed with Kraken [19], which uses exact alignments of raw shotgun sequences to k-mers derived from the resus macaque reference genome. Remaining reads were processed with Trimmomatic [20] to trim adapter sequences and low-quality ends (<Q20). Reads shorter than 35bp after trimming were discarded. rRNA sequences from all three domains of life were identified and removed with SortMeRNA 2.0 [21]. Contaminant sequences, like PhiX174 and sequencing primers, were removed with Bowtie2 [22].

#### Taxonomic profiling using MetaPhIAn

MetaPhIAn2 (Metagenomic Phylogenetic Analysis, version 2.0, [23]) was used for the taxonomic profiling of the metagenomic samples. Raw non-host reads were used directly, because low quality reads are ignored in addition to human, 16S rRNA and tRNA reads. MetaPhIAn2 includes and expanded set of ~1 million markers (184 ± 45 for each bacterial species) from > 16,000 reference genomes and > 7,500 unique species. Marker genes were identified for Bacteria, Archaea, viruses and eukaryotic microbes (Fungi and Protozoa) that are all crucial components of microbial communities.

#### Functional analysis

Filtered DNA sequences were mapped against a reference database of all proteins within the KEGG databases (version 75.0). The database is composed of 8,973,418 protein sequences extracted from 2,854 genomes. The search of translated DNA sequences was executed using Diamond [24] and hits that spanned ≥ 20 amino acids with ≥ 80% similarity were collected. In cases where one read matched these criteria against multiple proteins, only the protein or proteins (in the event of a tie) with the maximum bit score were collected. For pathway analysis, all proportional counts of KOs belonging to the pathway were summed for each pathway. Since one KO can be a member of multiple pathways (one-to-many relationship), this step increases the total proportional count in the pathway abundance table.

#### Alpha-diversity (within sample diversity) metrics

Richness is the sum of unique species found in each sample. Shannon diversity utilizes the richness of a sample along with the relative abundance of the present OTUs to calculate a diversity index.

#### Beta-diversity (sample-to-sample dissimilarity) metrics

All profiles are inter-compared in pair-wise fashion to determine a dissimilarity score and store it in a distance dissimilarity matrix. Distance functions produce low dissimilarity scores when comparing similar samples. Abundance-weighted sample pair-wise differences were calculated using the Bray-Curtis dissimilarity. Bray-Curtis dissimilarity is calculated by the ratio of the summed absolute differences in counts to the sum of abundances in the two samples [25].

#### Ordination, clustering and classification methods

Two-dimensional ordinations and hierarchical clustering maps of the samples in the form of dendrograms were created to graphically summarize the inter-sample relationships. Principal Coordinate Analysis (PCoA) is a method of two-dimensional ordination plotting that is used to help visualize complex relationships between samples. PCoA uses the sample-to-sample dissimilarity values to position the points relative to each other by maximizing the linear correlation between the dissimilarity values and the plot distances.

#### Whole microbiome significance testing

Permutational Analysis of Variance (PERMANOVA) is utilized for finding significant differences among discrete categorical or continuous variables. In this randomization/Monte Carlo permutation test, the samples are randomly reassigned to the various sample categories, and the between-category differences are compared to the true between-category differences. PERMANOVA utilizes the sample-to-sample distance matrix directly, not a derived ordination or clustering outcome.

#### Taxon, gene and pathway significance testing

The Wilcoxon Rank Sum test was employed to identify differentially abundant taxa, genes or pathways. Where samples could be paired across categories, a paired Wilcoxon Signed Rank test was employed. P-values were adjusted by Benjamini-Hochberg procedure to control for false discovery rates from multiple testing.

## Results

### Burn injury severely impacts overall health on post burn day 1

**Fig 1:**
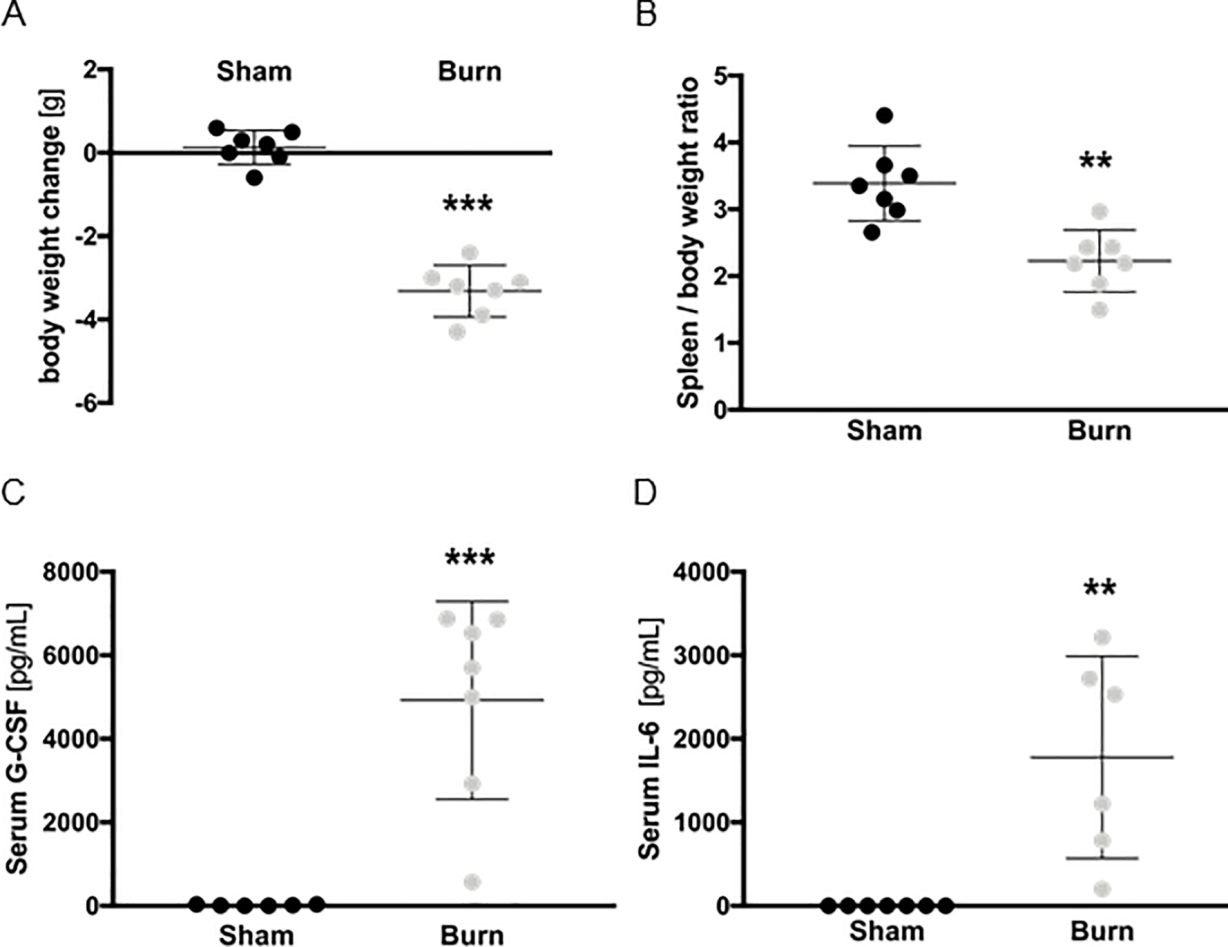
Burn injury severely impacts overall health on post burn day 1. Body weight (**A**) of mice was determined before and one day after burn or sham injury. Data are presented as weight change in g. Spleen-to-body weight ratios (B) were also determined. Serum G-CSF (**C**) and serum IL-6 (**D**) levels of the same mice were quantified by cytometric bead assay. For all experiments, each replicate is plotted (n = 7 mice/group), as well as mean ± SD. Asterisks indicate significant differences as assessed by unpaired, two-tailed Student’s t test: ** p < 0.01; ** p < 0.001.

Mice were subjected to a 28% full-thickness scald injury. To assess their overall health status after burn injury, we first assessed changes in body weight. On post burn day 1 (PBD1) mice had lost 3.3 g body weight on average, corresponding to approx. 10% of their total body mass (Fig 1A). We also analyzed spleen-to-body weight ratios, as the spleen is the biggest single secondary lymphoid organ in mice and of great immunological importance. Spleen-to-body weight ratios dropped significantly in burn mice compared to sham-treated controls (Fig 1B). To further assess the systemic immunological response, we quantified serum G-CSF and IL-6 levels. Both were significantly increased upon burn injury (Fig 1C and 1D).

### Taxonomic composition of the microbiome is altered upon burn injury

**Fig 2:**
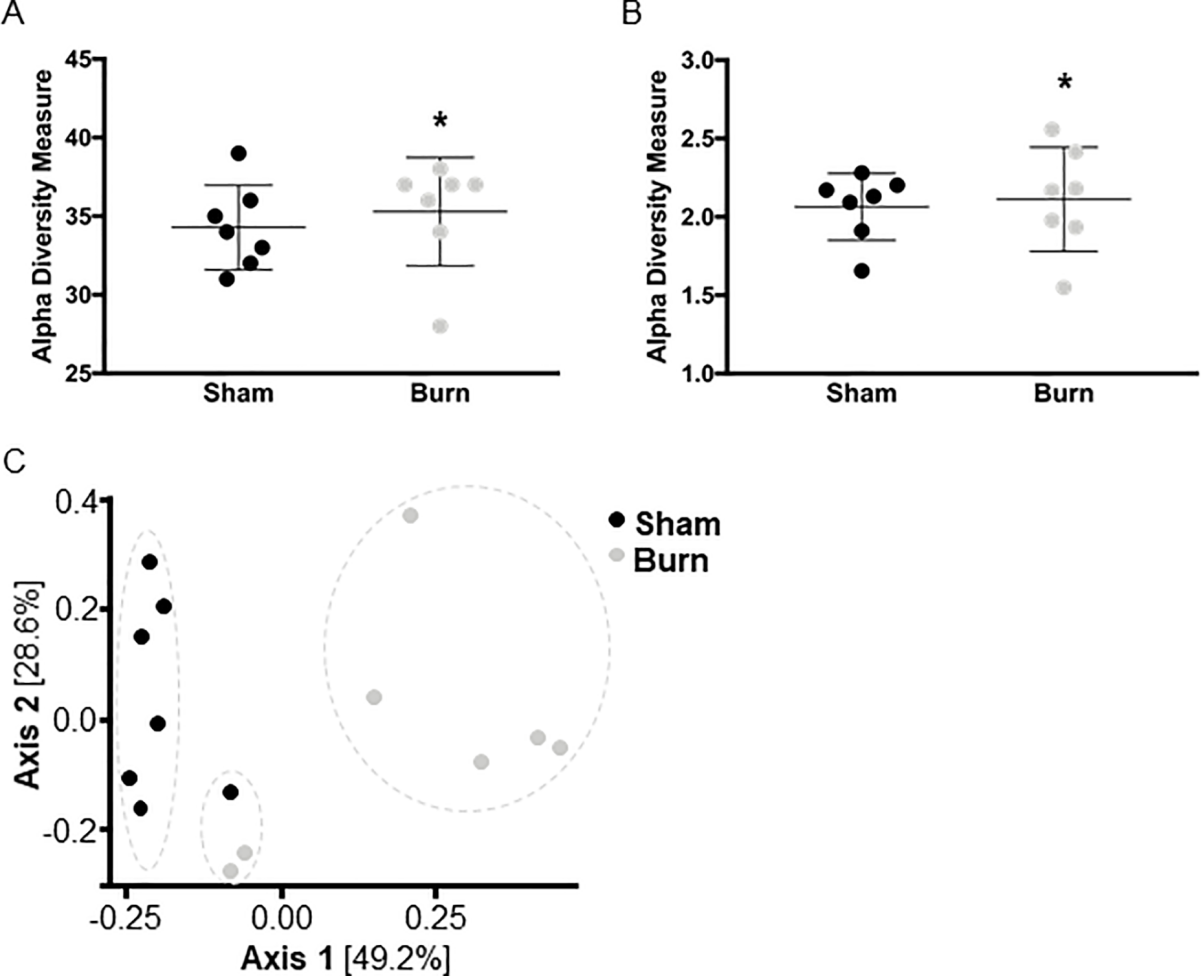
Microbiome structure upon burn injury. The richness (**A**) and Shannon index (**B**) of fecal samples collected from the cecum were used to estimate the level of diversity of the gut microbiome after burn injury (data shown as mean ± SD). Beta diversity was assessed by dimensional reduction of the Bray-Curtis distance between the microbiome samples, using the PCoA ordination method (**C**). For all experiments, each replicate is plotted (n = 7 mice/group), as well as mean ± SD. Asterisks indicate significant differences as assessed by Kruskal-Wallis rank sum test.

In order to analyze potential changes to the microbiome upon burn injury, cecal feces samples were collected on PBD1. The taxonomic richness (alpha diversity) was statistically similar between both groups: Similar sums of unique species were found in each sample (Fig 2A) and the relative abundance was similar as well (Fig 2B), indicating that the microbial community structure was not affected by burn injury. Nevertheless, beta diversity (sample-to-sample dissimilarity) was significantly altered between the two groups: Samples clustered together by injury type in the Bray-Curtis comparison, although one sham and two burn injury mice also clustered together (Fig 2C; p = 0.001, PERMANOVA).

**Fig 3:**
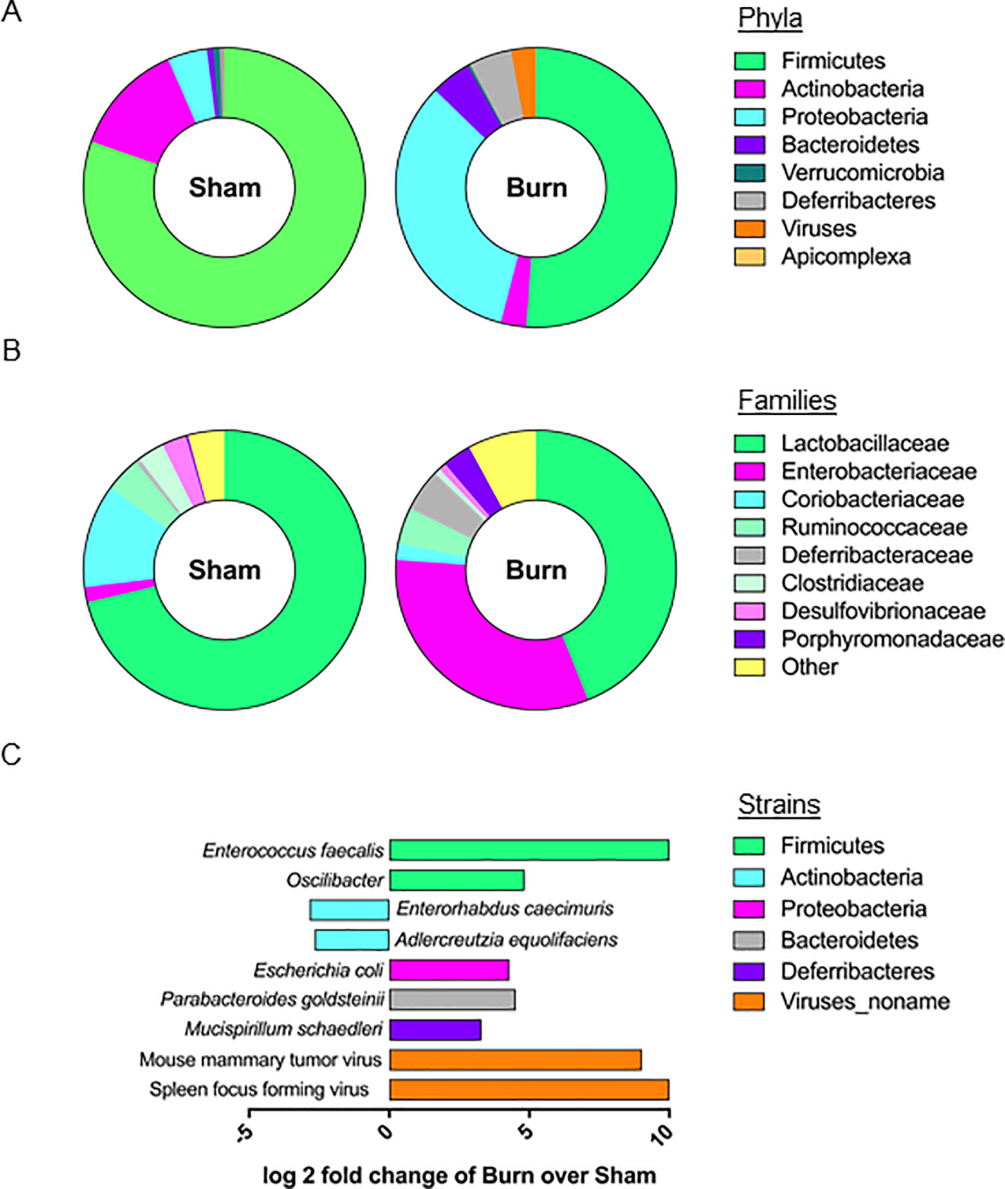
Taxonomic composition of the microbiome is altered upon burn injury. Sunburst plots show the most abundant taxa at the phylum (**A**) and family (**B**) level in the cecum (data shown as means). Differentially abundant strains (**C**) were considered significant if their FDR-corrected p-value was less than or equal to 0.05 and the absolute value of the log-2 fold change was greater or equal to 1. Points at either end of the x-axis indicate infinite log-2 fold change (the other groups mean abundance is 0). 59 strains were tested and only the 9 significantly different strains are displayed.

Regarding taxonomic composition, fecal microbial communities of control mice were dominated by *Firmicutes* (Fig 3A) on the phylum level. In burn mice *Firmicutes* and *Actinobacteria* were reduced, whereas *Proteobacteria*, *Deferribacteres*, *Bacteroidetes*, Viruses and *Apicomplexa* were enriched (Fig 3A). Out of the top eight phyla, only *Verrucomicrobia* was not significantly altered (Fig 3A). On the family level, *Enterobacteriaceae*, *Deferribacteraceae* and *Porphyromonadaceae* were enriched in the burn injury mice samples, while *Lactobacilaceae*, *Coriobacteriaceae*, *Clostridiaceae*, *Desulfovibrionaceae* were reduced (Fig 3B). *Ruminococcaceae* were not altered (Fig 3B). Regarding individual strains, *Adlercreutzia equolifaciens* and *Enterorhabdus caecimuris* were enriched in the sham injury mice samples, whereas *Parabacteroides goldsteinii*, *Mucispiillum schaedleri*, *Escherichia coli*, *Enterococcus faecalis*, *Oscillibacter* unclassified, Mouse mammary tumor virus (genus Betaretrovirus) and Spleen focus forming virus (genus Gammaretrovirus) were enriched in the burn injury mice samples (Fig 3C).

### 38 signaling pathways are differentially abundant upon burn injury

**Fig 4:**
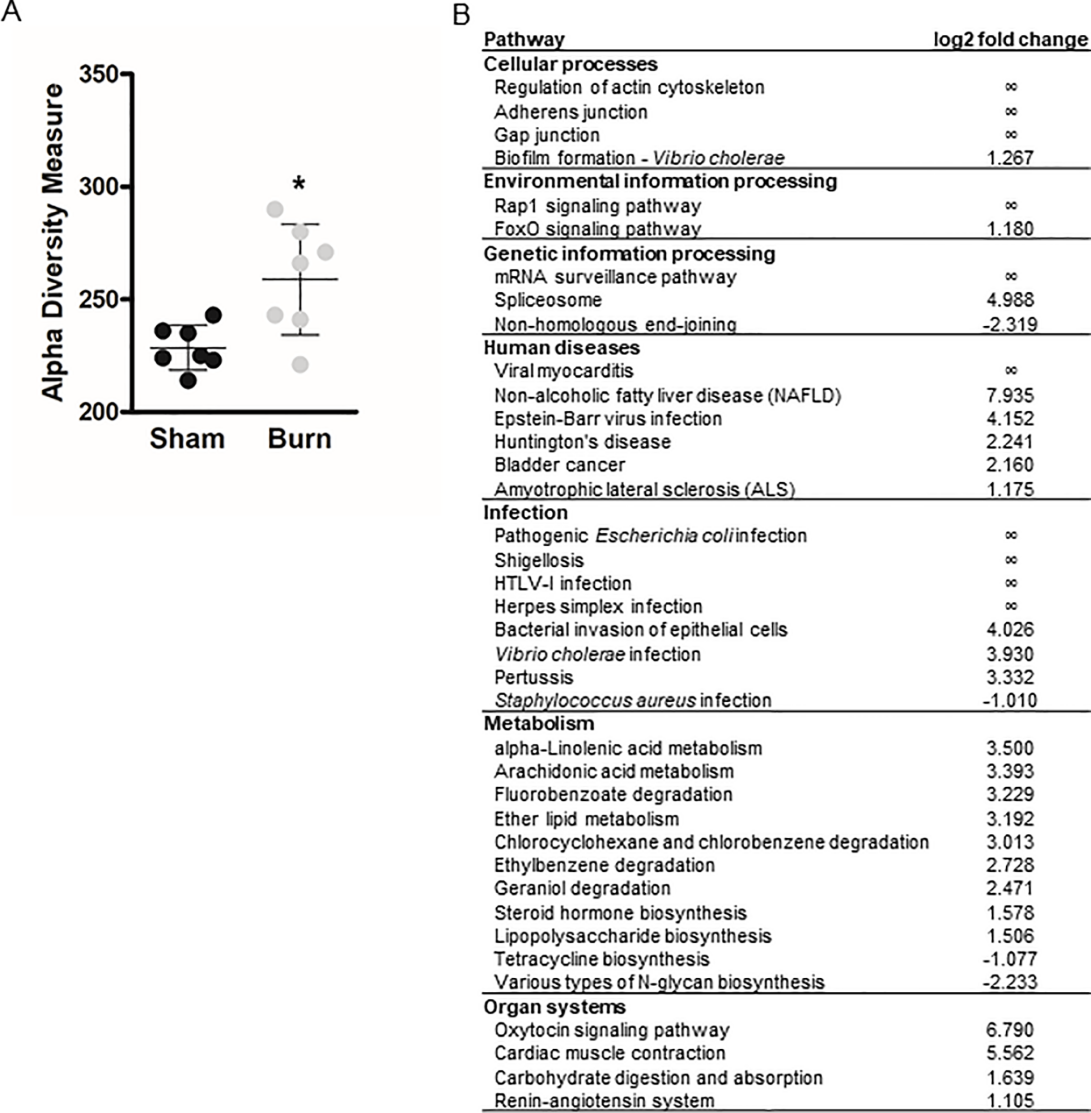
Pathway diversity is altered upon burn injury. The degree of variation of signaling pathways within a sample was assessed after burn or sham injury (**A**). Each replicate is presented, as well as mean ± SD. Asterisks indicate significant differences as assessed by Kruskal-Wallis rank sum test. Differentially abundant pathways in the cecal microbiome (**B**) were considered significant if their FDR-corrected p-value was less than or equal to 0.05 and the absolute value of the log-2 fold change was greater than or equal to 1. Infinite log-2 fold changes indicate that the other groups mean abundance is 0. Out of the 331 pathways tested, only the 38 pathways, whose abundance was significantly altered, are reported.

We determined pathway richness upon burn injury and observed a significant increase in alpha diversity metrics in burn mice (Fig 4A). We also determined the abundance of 331 signaling pathways: The abundance of 4 pathways was significantly reduced in burn injured mice, 34 were significantly enriched (Fig 4B). Altered pathways regulate cellular processes, environmental or genetic information processing and metabolism, or are associated with human diseases, infection or specific organ systems. Among the 38 altered pathways, 10 were not expressed in the microbiome of sham injured mice.

## Discussion

Previous work indicates that burn injury significantly alters the taxonomic composition of the microbiome [13] and that in mice, these changes were most pronounced one day after injury. Three days after burn, the dysbiosis had already decreased or normalized. We thus focused our analysis on PBD1. While the previous analysis addressed both the microbiome of the small and of the large intestine [13], we did not see increased permeability of the small intestine following burn injury [26]. Given that the cecum is the main site of fermentation and microbial-derived metabolite production in the mouse, changes to the cecal microbiome have the greatest potential to impact murine health. We thus focused our analyses on the cecal microbiome.

Similar to the previous report [13], we observed an overgrowth with normally low-abundant gram-negative bacteria of the *Proteobacteria*, *Deferribacteres* and *Bacteroidetes* phyla, compared to the normal predominance of gram-positive *Firmicutes*. The increase in *Proteobacteria* was the most pronounced change, particularly regarding the subfamily Enterobacteriaceae. This corresponds to the previously published work [13]. The Enterobacteriaeae family includes many opportunistic pathogens which are commonly found in septic patients, i.e. *Escherichia*, *Klebsiella*, *Proteus* and *Citrobacter* [27], but it not yet known which individual strains of these bacteria elicit systemic inflammation after burn injury. We observed an increase in the abundance of *Enterococcus faecalis*, *Oscilibacter*, *Escherichia coli*, *Parabacteroides goldsteinii* and *Mucispirillum schaedleri* upon burn injury in our study. Enterococci are the third leading cause of nosocomical bloodstream infections, among which 60% are caused by *E*. *faecalis* [28]. Next to *Escherichia*, which is commonly found in septic patients [27], this identifies *E*. *faecalis* as a strong candidate which may induce systemic inflammation after burn. Individual reports of bacteraemia also exist for *Oscilibacter* [29] and *P*. *goldsteinii* [30]. Further studies will be necessary to determine which strains are the culprits in septic burn patients.

We also noted decreases in potentially protective bacteria. The majority of butyrate producing bacteria belong to the *Firmicutes* phylum, which was significantly less abundant in the burn injury mice. Regarding individual strains, the butyrate producer *Enterorhabdus caecimuris* was less abundant in the burn injury mice as well. Butyrate is well known for regulating T-cell function and the systemic immune response [31]. We have previously shown that colonic butyrate levels decrease following burn injury [26] and that butyrate reduces T cell death in an acid sphingomyelinase-dependent manner [32]. T cell depletion is a key component in burn-induced immunosuppression and enhanced susceptibility to opportunistic infections. Thus, butyrate restoration is a potential new therapy to ameliorate immunosuppression in burn. Indeed, we have previously shown that fecal microbiota transplants that reconstituted butyrate producers to burn injured mice ameliorate burn-induced colon permeability [26]. Taken together, our data motivates further research into probiotic supplementation with butyrate producing bacteria as a novel treatment for burn patients to hopefully prevent sepsis.

To our knowledge, our study is the first to assess functional gene expression of the microbiome upon burn injury, to determine the microbiome’s physiological capabilities for overgrowth and pathogenic invasion. Pathway richness was significantly increased upon burn injury and the abundance of 38 pathways were significantly different between scald and sham mice. Among these, 9 metabolic pathways were significantly upregulated and 2 significantly suppressed.

Tetracycline biosynthesis was one of the suppressed pathways. Tetracyclines are antibiotics produced by *Streptomyces* (*Actinobacteriae*). A reduced abundance of this pathway corresponds to the reduced abundance of *Actinobacteriae* in burn injured mice and constitutes a loss of a self-regulation of the microbiome. The second downregulated pathway, N-glycan biosynthesis, can result in immune evasion, as non-glycosylated bacterial proteins are often no longer recognized by the host immune system [33].

Many of the metabolic pathways that were more abundant in the microbiome of burn injury mice have potential immunomodulatory consequences: Increased production of prostaglandin E2 –either through increased biosynthesis of its precursor alpha-linoleic acid, through increased arachidonic-acid biosynthesis or through increased ether lipid metabolism—suppresses the type 1 immune response [34].

LPS biosynthesis was also more abundant upon burn injury. This matches the expansion of *Bacteriodetes* found in burn injury microbiome samples. *Bacteriodetes* are major producers of LPS. *Bacteriodetes*-derived LPS has been reported to be immunoinhibitory, which may contribute to disease susceptibility upon burn injury [35]. Additionally, the metabolome of the gut microbiome has been previously shown to be predictive of host dysbiosis [36]. LPS biosynthesis was among the most predictive pathways, as was Chlorocyclohexane and chlorobenzene degradation, which was also significantly more abundant upon burn injury [36].

A large number of significantly affected pathways upon burn injury are linked to infection and pathogenic invasion. Particularly the pathways regulating the actin cytoskeleton, gap- and adherens junctions and bacterial invasion of epithelial cells would contribute to the disruption of the intestinal barrier and enable the translocation of bacteria and subsequent sepsis.

Our data confirms taxonomic differences to the intestinal microbiome upon burn injury, particularly the overgrowth of *Enterobacteriaceae*. We also identify functional gene expression changes upon burn injury, linking particular pathways to dysbiosis, lack of barrier integrity and pathogenic invasion for the first time. The disruption of the intestinal barrier following dysbiosis and the resulting translocation of bacteria and their products from the gut lumen to the circulation can lead to (Gram-negative) bacteremia, resulting in sepsis, multi-organ failure and even death of burn patients.

## Supporting information

S1 Table Burn injury severely impacts overall health on post burn day 1—Supplement to Fig 1. Table 1 includes individual data points for body weight before burn and on PBD1, spleen weight, spleen-to-body weight ratio, serum G-CSF and serum IL-6 levels for each sample.
(XLS)

S2 Table Microbiome structure upon burn injury—Supplement to Fig 2. Table 2 includes individual data points on richness, Shannon index and dissimilarity matrix for beta diversity measures.
(XLS)

S3 Table Taxonomic composition of the microbiome is altered upon burn injury—Supplement to Fig 3. Table 3 includes all count tables and the mean abundance values for the different phyla, families and strains.
(XLS)

S4 Table Pathway diversity is altered upon burn injury—Supplement to Fig 4. Table 4 includes individual data points on pathway alpha diversity and count tables for pathways and individual genes, as well as the mean log2 fold change values for each gene and pathway.
(XLS)

## References

[1] Hanschen M, Tajima G, O'Leary F, Ikeda K, Lederer JA. Injury induces early activation of T-cell receptor signaling pathways in CD4+ regulatory T cells. Shock (Augusta, Ga). 2011;35(3):252–7. Epub 2010/08/20. 10.1097/SHK.0b013e3181f489c5 ; PubMed Central PMCID: PMCPMC3045756. 20720513 PMC3045756

[2] MacConmara MP, Tajima G, O'Leary F, Delisle AJ, McKenna AM, Stallwood CG, et al Regulatory T cells suppress antigen-driven CD4 T cell reactivity following injury. Journal of leukocyte biology. 2011;89(1):137–47. Epub 2010/10/05. 10.1189/jlb.0210082 ; PubMed Central PMCID: PMCPMC3004517. 20884652 PMC3004517

[3] Magnotti LJ, Deitch EA. Burns, bacterial translocation, gut barrier function, and failure. The Journal of burn care & rehabilitation. 2005;26(5):383–91. Epub 2005/09/10. . 16151282 10.1097/01.bcr.0000176878.79267.e8

[4] Lichtman SM. Bacterial [correction of baterial] translocation in humans. Journal of pediatric gastroenterology and nutrition. 2001;33(1):1–10. Epub 2001/08/02. . 11479400 10.1097/00005176-200107000-00001

[5] Van Leeuwen PA, Boermeester MA, Houdijk AP, Ferwerda CC, Cuesta MA, Meyer S, et al Clinical significance of translocation. Gut. 1994;35(1 Suppl):S28–34. Epub 1994/01/01. ; PubMed Central PMCID: PMCPMC1378143. 8125386 10.1136/gut.35.1_suppl.s28 PMC1378143

[6] Honda K, Littman DR. The microbiome in infectious disease and inflammation. Annual review of immunology. 2012;30:759–95. Epub 2012/01/10. 10.1146/annurev-immunol-020711-074937 ; PubMed Central PMCID: PMCPMC4426968. 22224764 PMC4426968

[7] Round JL, Mazmanian SK. The gut microbiota shapes intestinal immune responses during health and disease. Nature reviews Immunology. 2009;9(5):313–23. Epub 2009/04/04. 10.1038/nri2515 ; PubMed Central PMCID: PMCPMC4095778. 19343057 PMC4095778

[8] Barcenilla A, Pryde SE, Martin JC, Duncan SH, Stewart CS, Henderson C, et al Phylogenetic relationships of butyrate-producing bacteria from the human gut. Applied and environmental microbiology. 2000;66(4):1654–61. Epub 2000/04/01. ; PubMed Central PMCID: PMCPMC92037. 10742256 10.1128/aem.66.4.1654-1661.2000 PMC92037

[9] Cummings JH, Macfarlane GT. Colonic microflora: nutrition and health. Nutrition (Burbank, Los Angeles County, Calif). 1997;13(5):476–8. Epub 1997/05/01. . 9225346 10.1016/s0899-9007(97)00114-7

[10] Bien J, Palagani V, Bozko P. The intestinal microbiota dysbiosis and Clostridium difficile infection: is there a relationship with inflammatory bowel disease? Therapeutic advances in gastroenterology. 2013;6(1):53–68. Epub 2013/01/16. 10.1177/1756283X12454590 ; PubMed Central PMCID: PMCPMC3539291. 23320050 PMC3539291

[11] Guarner F, Malagelada JR. Gut flora in health and disease. Lancet (London, England). 2003;361(9356):512–9. Epub 2003/02/14. 10.1016/s0140-6736(03)12489-0 . 12583961

[12] Xavier RJ, Podolsky DK. Unravelling the pathogenesis of inflammatory bowel disease. Nature. 2007;448(7152):427–34. Epub 2007/07/27. 10.1038/nature06005 . 17653185

[13] Earley ZM, Akhtar S, Green SJ, Naqib A, Khan O, Cannon AR, et al Burn Injury Alters the Intestinal Microbiome and Increases Gut Permeability and Bacterial Translocation. PloS one. 2015;10(7):e0129996 Epub 2015/07/15. 10.1371/journal.pone.0129996 ; PubMed Central PMCID: PMCPMC4496078. 26154283 PMC4496078

[14] Gill SR, Pop M, Deboy RT, Eckburg PB, Turnbaugh PJ, Samuel BS, et al Metagenomic analysis of the human distal gut microbiome. Science (New York, NY). 2006;312(5778):1355–9. Epub 2006/06/03. 10.1126/science.1124234 ; PubMed Central PMCID: PMCPMC3027896. 16741115 PMC3027896

[15] Turnbaugh PJ, Ley RE, Hamady M, Fraser-Liggett CM, Knight R, Gordon JI. The human microbiome project. Nature. 2007;449(7164):804–10. Epub 2007/10/19. 10.1038/nature06244 ; PubMed Central PMCID: PMCPMC3709439. 17943116 PMC3709439

[16] Tschop J, Martignoni A, Reid MD, Adediran SG, Gardner J, Noel GJ, et al Differential immunological phenotypes are exhibited after scald and flame burns. Shock (Augusta, Ga). 2009;31(2):157–63. Epub 2008/07/25. 10.1097/SHK.0b013e31817fbf4d ; PubMed Central PMCID: PMCPMC2674561. 18650781 PMC2674561

[17] Moss NM, Gough DB, Jordan AL, Grbic JT, Wood JJ, Rodrick ML, et al Temporal correlation of impaired immune response after thermal injury with susceptibility to infection in a murine model. Surgery. 1988;104(5):882–7. Epub 1988/11/01. . 3263707

[18] Faunce DE, Llanas JN, Patel PJ, Gregory MS, Duffner LA, Kovacs EJ. Neutrophil chemokine production in the skin following scald injury. Burns: journal of the International Society for Burn Injuries. 1999;25(5):403–10. Epub 1999/08/10. . 10439148 10.1016/s0305-4179(99)00014-5

[19] Wood DE, Salzberg SL. Kraken: ultrafast metagenomic sequence classification using exact alignments. Genome biology. 2014;15(3):R46 Epub 2014/03/04. 10.1186/gb-2014-15-3-r46 ; PubMed Central PMCID: PMCPMC4053813. 24580807 PMC4053813

[20] Bolger AM, Lohse M, Usadel B. Trimmomatic: a flexible trimmer for Illumina sequence data. Bioinformatics (Oxford, England). 2014;30(15):2114–20. Epub 2014/04/04. 10.1093/bioinformatics/btu170 ; PubMed Central PMCID: PMCPMC4103590. 24695404 PMC4103590

[21] Kopylova E, Noe L, Touzet H. SortMeRNA: fast and accurate filtering of ribosomal RNAs in metatranscriptomic data. Bioinformatics (Oxford, England). 2012;28(24):3211–7. Epub 2012/10/17. 10.1093/bioinformatics/bts611 . 23071270

[22] Langmead B, Salzberg SL. Fast gapped-read alignment with Bowtie 2. Nature methods. 2012;9(4):357–9. Epub 2012/03/06. 10.1038/nmeth.1923 ; PubMed Central PMCID: PMCPMC3322381. 22388286 PMC3322381

[23] Truong DT, Franzosa EA, Tickle TL, Scholz M, Weingart G, Pasolli E, et al MetaPhlAn2 for enhanced metagenomic taxonomic profiling. Nature methods. 2015;12(10):902–3. Epub 2015/09/30. 10.1038/nmeth.3589 . 26418763

[24] Buchfink B, Xie C, Huson DH. Fast and sensitive protein alignment using DIAMOND. Nature methods. 2015;12(1):59–60. Epub 2014/11/18. 10.1038/nmeth.3176 . 25402007

[25] Bray JR, Curtis JT. An Ordination of the Upland Forest Communities of Southern Wisconsin. Ecological Monographs. 1957;27(4):325–49. 10.2307/1942268

[26] Kuethe JW, Armocida SM, Midura EF, Rice TC, Hildeman DA, Healy DP, et al Fecal Microbiota Transplant Restores Mucosal Integrity in a Murine Model of Burn Injury. Shock (Augusta, Ga). 2016;45(6):647–52. Epub 2015/12/20. 10.1097/shk.0000000000000551 ; PubMed Central PMCID: PMCPMC5103310. 26682948 PMC5103310

[27] MacFie J, O'Boyle C, Mitchell CJ, Buckley PM, Johnstone D, Sudworth P. Gut origin of sepsis: a prospective study investigating associations between bacterial translocation, gastric microflora, and septic morbidity. Gut. 1999;45(2):223–8. Epub 1999/07/14. ; PubMed Central PMCID: PMCPMC1727620. 10403734 10.1136/gut.45.2.223 PMC1727620

[28] Bar K, Wisplinghoff H, Wenzel RP, Bearman GM, Edmond MB. Systemic inflammatory response syndrome in adult patients with nosocomial bloodstream infections due to enterococci. BMC infectious diseases. 2006;6:145 Epub 2006/09/28. 10.1186/1471-2334-6-145 ; PubMed Central PMCID: PMCPMC1592497. 17002792 PMC1592497

[29] Sydenham TV, Arpi M, Klein K, Justesen US. Four cases of bacteremia caused by Oscillibacter ruminantium, a newly described species. Journal of clinical microbiology. 2014;52(4):1304–7. Epub 2014/02/07. 10.1128/JCM.03128-13 ; PubMed Central PMCID: PMCPMC3993470. 24501034 PMC3993470

[30] Awadel-Kariem FM, Patel P, Kapoor J, Brazier JS, Goldstein EJ. First report of Parabacteroides goldsteinii bacteraemia in a patient with complicated intra-abdominal infection. Anaerobe. 2010;16(3):223–5. Epub 2010/02/09. 10.1016/j.anaerobe.2010.01.001 . 20139022

[31] Furusawa Y, Obata Y, Fukuda S, Endo TA, Nakato G, Takahashi D, et al Commensal microbe-derived butyrate induces the differentiation of colonic regulatory T cells. Nature. 2013;504(7480):446–50. Epub 2013/11/15. 10.1038/nature12721 . 24226770

[32] Rice TC, Armocida SM, Kuethe JW, Midura EF, Jain A, Hildeman DA, et al Burn injury influences the T cell homeostasis in a butyrate-acid sphingomyelinase dependent manner. Cellular immunology. 2017;313:25–31. Epub 2017/01/09. 10.1016/j.cellimm.2016.12.004 ; PubMed Central PMCID: PMCPMC5559081. 28063598 PMC5559081

[33] Nothaft H, Szymanski CM. Bacterial protein N-glycosylation: new perspectives and applications. The Journal of biological chemistry. 2013;288(10):6912–20. Epub 2013/01/19. 10.1074/jbc.R112.417857 ; PubMed Central PMCID: PMCPMC3591601. 23329827 PMC3591601

[34] Namazi MR. The beneficial and detrimental effects of linoleic acid on autoimmune disorders. Autoimmunity. 2004;37(1):73–5. Epub 2004/04/30. . 15115315 10.1080/08916930310001637968

[35] d'Hennezel E, Abubucker S, Murphy LO, Cullen TW. Total Lipopolysaccharide from the Human Gut Microbiome Silences Toll-Like Receptor Signaling. mSystems. 2017;2(6). Epub 2017/11/21. 10.1128/mSystems.00046-17 ; PubMed Central PMCID: PMCPMC5686520. 29152585 PMC5686520

[36] Larsen PE, Dai Y. Metabolome of human gut microbiome is predictive of host dysbiosis. GigaScience. 2015;4:42 Epub 2015/09/18. 10.1186/s13742-015-0084-3 ; PubMed Central PMCID: PMCPMC4570295. 26380076 PMC4570295

